# From Environmental Threat to Control: A Review of Technologies for Removal of Quaternary Ammonium Compounds from Wastewater

**DOI:** 10.3390/membranes16010001

**Published:** 2025-12-19

**Authors:** Aleksandra Klimonda, Izabela Kowalska

**Affiliations:** Faculty of Environmental Engineering, Wroclaw University of Science and Technology, Wybrzeże S. Wyspiańskiego 27, 50-370 Wrocław, Poland

**Keywords:** quaternary ammonium compounds, micropollutants, forward osmosis, cationic surfactants, emerging contaminants, membrane separation technology, environmental pollution control

## Abstract

Cationic surfactants from the group of quaternary ammonium compounds (QACs) are widely used in disinfectants, cosmetics, and household and industrial products. Their strong antimicrobial activity and chemical stability make them valuable in applications but also highly persistent and toxic when released into aquatic environments. This problem has become increasingly relevant during and after the COVID-19 pandemic, when global use of QAC-based disinfectants increased drastically, resulting in their frequent detection in municipal, hospital, and industrial effluents. The concentrations of QACs reported in wastewater range from trace levels to several mg/L, often reaching inhibitory thresholds for biological treatment processes. Although surfactants are not listed in any current European directive, the revised Directive (EU) 2024/1440 classifies micropollutants as a priority group, imposing stricter environmental quality standards and mandatory monitoring requirements. Within this regulatory framework, QACs are recognized as compounds of emerging concern, and their effective removal from wastewater has become a critical challenge. This review summarizes the current knowledge on conventional treatment technologies (coagulation, adsorption, ion exchange, advanced oxidation, and biological processes) and membrane-based methods (ultrafiltration, nanofiltration, reverse osmosis, forward osmosis, and hybrid systems) for the removal of cationic surfactants from water and wastewater. Mechanisms of separation, performance, and operational limitations are discussed.

## 1. Introduction

Micropollutants such as pharmaceuticals, pesticides, and surfactants represent an emerging challenge for water quality and safety worldwide [1]. These anthropogenic compounds are typically persistent, biologically active, and poorly removed by conventional wastewater treatment, leading to their accumulation in the aquatic environment. Among them, cationic surfactants (CSs), particularly quaternary ammonium compounds (QACs), have attracted growing concern due to their widespread use, strong antimicrobial activity, and toxicity to aquatic organisms. Their environmental relevance has recently been amplified by two converging factors: the unprecedented surge in disinfectant consumption during and after the COVID-19 pandemic and the adoption of Directive (EU) 2024/1440 [2], which strengthens regulatory control of micropollutants in European waters. Together, these developments highlight the urgent need to evaluate and improve available treatment technologies for the removal of QACs from water and wastewater.

Although several studies have investigated the fate and removal of surfactants in water treatment, most reviews have focused on anionic and nonionic compounds, whereas cationic surfactants have received comparatively little attention. Among these, quaternary ammonium compounds (QACs) are particularly persistent and hazardous, exhibiting strong interactions with negatively charged particles, natural organic matter, and microbial communities. Despite their growing environmental relevance, a comprehensive synthesis critically comparing the performance of conventional and membrane-based treatment technologies for their removal is still lacking. This review aims to address this gap by summarizing the current state of knowledge on treatment approaches for cationic surfactants. It highlights removal mechanisms, treatment efficiency, limitations of individual methods, and prospects for future development under increasingly stringent regulatory frameworks.

This review provides a comprehensive overview of the physicochemical properties, environmental occurrence, and ecological implications of cationic surfactants. It then critically examines conventional treatment strategies, including coagulation, adsorption, ion exchange, advanced oxidation, and biological processes. Particular attention is given to pressure-driven membrane technologies (ultrafiltration, nanofiltration, and reverse osmosis), forward osmosis, and hybrid systems. The discussion concludes with an integrated assessment of the advantages, limitations, and future directions of these approaches in the context of evolving regulatory and environmental requirements.

## 2. Cationic Surfactants: Properties, Occurrence, and Environmental Relevance

### 2.1. Chemical Structure and Physicochemical Properties

Surfactants are amphiphilic compounds comprising a hydrophobic tail and a hydrophilic head group, which enables their adsorption at interfaces. Depending on the charge of the hydrophilic moiety, surfactants are traditionally classified into four categories: anionic (AS), cationic (CS), nonionic (NS), and amphoteric or zwitterionic (ZS) types.

**Figure 1:**
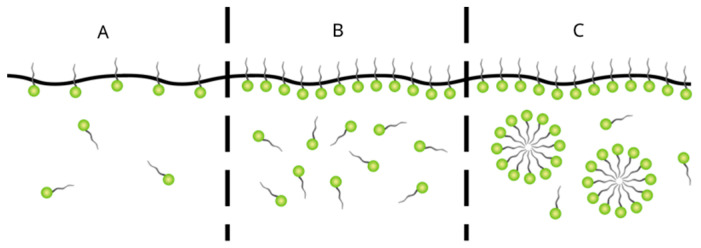
Schematic representation illustrating surfactant behavior in aqueous solution: (**A**) monomers at low concentrations, (**B**) adsorption at the air–water interface as an interfacial monolayer, and (**C**) micelle formation above the critical micelle concentration (CMC).

**Figure 2:**
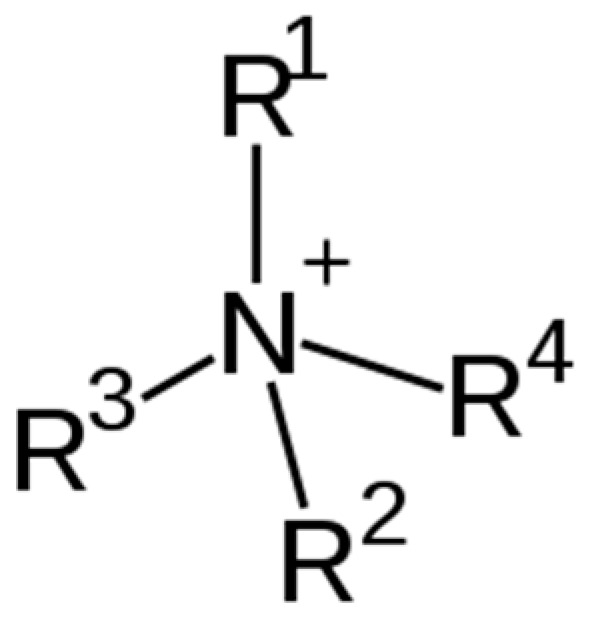
Quaternary nitrogen atom in the polar head group of cationic surfactants.

A common feature of surfactants, arising from their amphiphilic molecular structure, is their surface activity, expressed as a reduction in surface and interfacial tension [3]. At phase boundaries, surfactant molecules adsorb with their polar heads oriented toward the aqueous phase, forming interfacial films that stabilize otherwise immiscible systems (Figure 1). At low concentrations, surfactants exist mainly as monomers, while increasing concentration drives aggregation: first in the form of interfacial monolayers (Figure 1B), and above the critical micelle concentration (CMC) as micelles of colloidal dimensions (Figure 1C) [4,5]. The evolution of these structures in aqueous solution is illustrated in Figure 2.

Table 1 summarizes the representative aggregation numbers (n_a_g) and molecular weights of surfactant micelles, indicating that each micelle may contain tens to hundreds of monomers. Such a substantial increase in size and molecular weight relative to individual molecules is critical for understanding surfactant behavior during purification and removal processes.

Cationic surfactants are characterized by a positively charged headgroup, which strongly interacts with negatively charged surfaces, cell membranes, and natural organic matter. Unlike anionic surfactants, which dominate the detergent and household product market [9], CSs constitute a smaller but environmentally critical fraction due to their permanent positive charge and associated toxicity [10]. The most important subclass of cationic surfactants is quaternary ammonium compounds (QACs), in which the nitrogen atom carries (Figure 2) a permanent positive charge, typically balanced by a chloride or bromide counter-ion. QACs, such as benzalkonium chloride (BAC), cetyltrimethylammonium bromide (CTAB), and cetylpyridinium chloride (CPC), are widely used as disinfectants, preservatives, and phase-transfer catalysts [11,12,13].

The antimicrobial activity of QACs is primarily associated with their interaction with microbial cell membranes. Positively charged QAC molecules adsorb onto negatively charged cell surfaces and, through hydrophobic interactions, penetrate the phospholipid bilayer. This disrupts membrane integrity, increases permeability, and causes leakage of intracellular components, ultimately leading to cell death [14,15]. The efficiency of this mechanism depends mainly on the length of the alkyl chain and the molecular structure (QACs with C_12_–C_16_ chains show the highest activity, particularly against Gram-positive bacteria). Moreover, QACs exhibit strong virucidal properties against lipid-enveloped viruses, including SARS-CoV-2, by destabilizing and solubilizing the viral lipid envelope [16].

### 2.2. History and Development

QACs represent one of the earliest classes of modern disinfectants. Their discovery dates back to the early 20th century, when Jacobs and his collaborators [17] demonstrated the germicidal properties of quaternary salts of hexamethylenetetramine and related derivatives. Less than two decades later, Gerhard Domagk, who was later awarded the Nobel Prize, introduced benzalkonium chloride (BAC), which soon became one of the most widely applied representatives of this group [18]. BAC was the first cationic surfactant approved by the United States Environmental Protection Agency (US EPA) in 1947 [19]. Between the 1950s and 1970s, interest in QACs increased rapidly, reflecting their widespread adoption in domestic, agricultural, industrial, and clinical applications.

Following this expansion, it became increasingly evident that traditional QACs, such as BAC, posed environmental concerns because of their persistence and ecotoxicity, which accelerated the search for more biodegradable alternatives. Although the concept of ester-containing quaternary ammonium compounds dates back several decades, their practical application began much later. The first patents describing triethanolamine-based esterquats intended for fabric softeners were filed in the late 1970s [20] and large-scale production followed in the 1980s [21]. This recognition led in the 1980s to the development of biodegradable quaternary ammonium compounds containing ester linkages (esterquats). In contrast to classical disinfectant-type QACs, a new class of CSs was not designed for antimicrobial purposes but for fabric conditioning, as their cationic and hydrophobic structure effectively reduces fiber friction and static charge [22]. Representative examples such as TEAQ, DEEDMAC, and DHTDMAC quickly became industry standards and were later incorporated into cosmetic and personal care formulations [23].

By the 2010s, QACs were increasingly associated with antimicrobial resistance and ecological risks. Their use peaked during the COVID-19 pandemic, when the massive application of QAC-based disinfectants led to elevated concentrations in wastewater and surface waters. According to the U.S. EPA List N (disinfectants approved for use against SARS-CoV-2), among the 629 products currently registered, nearly 300 contain QACs as active ingredients [24].

Around the 1990s, another group of cationic surfactants known as gemini surfactants was identified [25]. These compounds contain two hydrophilic headgroups and two hydrophobic tails connected by a short spacer, which gives them higher surface activity and much lower critical micelle concentrations compared to conventional surfactants [26]. Gemini QACs attracted attention due to their strong antimicrobial properties and high efficiency at low doses [27,28,29]. However, their increased hydrophobicity and limited biodegradability raise environmental concerns that so far restrict their large-scale application [27].

Although quaternary ammonium compounds constitute the dominant group of cationic surfactants, several non-ammonium types such as pyridinium, imidazolinium, and phosphonium surfactants are also known [30,31,32]. These compounds share similar electrostatic and surface-active properties but differ in their cationic centres and are mainly used as antiseptics, antistatic agents, and corrosion inhibitors rather than general disinfectants [33].

**Figure 3:**
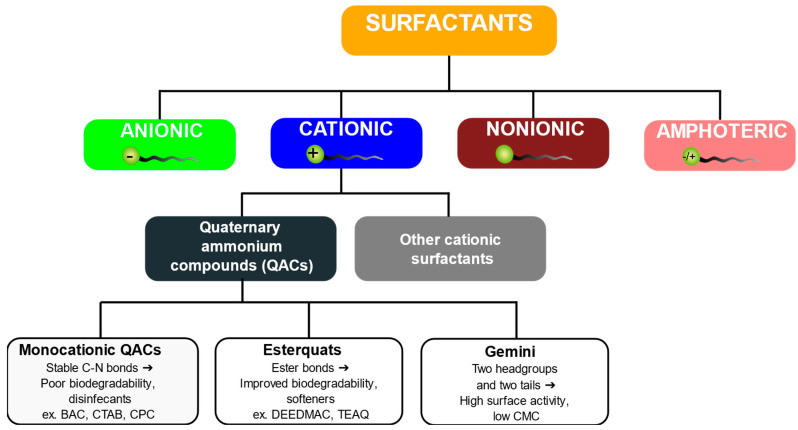
Classification of surfactants and quaternary ammonium compounds.

Currently, CSs are considered a double-edged sword: on the one hand, they are indispensable biocides; on the other hand, they pose a serious threat to the environment. Recent research has focused on the design of safer and more sustainable alternatives, including next-generation esterquats, amino acid–based surfactants, and biosurfactants. These approaches aim to balance antimicrobial efficacy with improved biodegradability and reduced ecological impact. To illustrate the position of QACs among other surfactant classes and highlight their structural variants, a schematic overview is presented in Figure 3.

Cationic surfactants are among the most versatile surface-active agents used worldwide. Their unique ability to adsorb onto negatively charged surfaces makes them indispensable in a wide spectrum of products, from disinfectants, sanitisers, and household cleaners to textile conditioners, cosmetics, and pharmaceutical formulations. Classical QACs such as benzalkonium chloride (BAC), cetylpyridinium chloride (CPC), and cetyltrimethylammonium bromide (CTAB) dominate hygiene, disinfection, and industrial applications, while modern esterquats (e.g., triethanolamine esterquat—TEAQ, di(ethoxyethyl) dimethyl ammonium chloride—DEEDMAC) have become the standard in fabric softeners and personal care products due to their improved biodegradability and lower aquatic toxicity. In addition to their established roles in healthcare and domestic use, QACs also find applications in aquaculture, agriculture, and food processing sanitation, reflecting their broad antimicrobial spectrum. The COVID-19 pandemic further boosted the global demand for these compounds, particularly as antiviral and disinfecting agents in hygiene products. Representative examples of applications for both groups are summarized in Table 2.

Recent market analyses indicate that the global QAC market was valued at approximately USD 1.4 billion in 2025 and is estimated to grow at a CAGR of around 5.19% between 2025 and 2034 [34]. In contrast, the esterquat market reached approximately USD 2.4 billion in 2023, growing at 10% annually [35], largely driven by its widespread use in high-volume consumer products such as fabric softeners and personal care formulations. Within the application mix, disinfectants represented approximately 41 to 42% of total demand, while fabric softeners, surfactants, antistatic agents, and others constituted the remaining [34]. These trends underscore the increasing industrial and economic significance of cationic surfactants in multiple sectors.

### 2.3. Sources and Occurrence of Cationic Surfactants

The widespread use of CSs in household, institutional, and industrial products leads to their occurrence in municipal, hospital, and industrial wastewaters. Their concentrations vary considerably depending on the source of wastewater, with the highest levels typically observed in industrial effluents [36,37,38]. Elevated CS content is also found in laundry and hospital wastewaters [38,39,40], whereas municipal influents generally contain lower concentrations [41]. Representative data on QAC concentrations in different wastewater sources are summarized in Table 3.

Once released from these sectors, cationic surfactants are continuously discharged into sewage systems. Influent concentrations of QACs in municipal wastewater commonly reach tens of µg/L, whereas concentrations in treated effluents from wastewater treatment plants (WWTPs) are generally below 10 µg/L, reflecting high (>90%) but incomplete removal efficiencies [43]. During the COVID-19 pandemic, Hladik et al. [44] reported that QACs were detected in 100% of WWTP effluent samples at concentrations ranging from 0.04 to 3.5 µg L^−1^, occasionally exceeding the predicted no-effect concentration (PNEC = 0.415 µg/L).

Although most QACs are generally efficiently removed during wastewater treatment, residual emissions and occasional untreated discharges result in their measurable presence in surface waters and sediments or other media [41,42,43,44,45,46,47,48]. Representative concentrations of QACs in wastewater, sludge, surface waters, sediments, and soils reported in recent studies are summarized in Table 4.

The detection of QACs in river and lake sediments provides clear evidence of their environmental persistence and historical accumulation. Analyses of sediment cores revealed distinct temporal trends in QAC accumulation. In lakes receiving mixed domestic and industrial wastewater (Lake Pepin and Duluth Harbor), peak concentrations occurred in the 1980s, followed by a decline associated with improved treatment and source control. Similar results were reported by Lennartz et al. [49], who found the maximum sum of QAC concentrations in sediments of the Lahn River (Germany) corresponding to deposits from approximately 1983–1984. The long-term presence of QACs in sediments indicates that these compounds can persist for decades once introduced into the environment [45].

In addition to their accumulation in natural sediments, QACs are also extensively retained within wastewater treatment systems. During biological treatment, a substantial portion of these compounds adsorbs onto activated sludge flocs [47], leading to elevated concentrations in sewage sludge and raising concerns about their fate during sludge handling and disposal. The mechanisms governing their retention and removal are discussed in Section 3.3.

Beyond aquatic systems, QACs are now frequently detected in indoor environments, indicating direct and continuous human exposure. During the COVID-19 pandemic, the sum of 18 QACs in residential dust from homes and public areas in South China reached 150 µg/g, with a median concentration of 42.2 µg/g [50]. Such findings underscore the widespread nature of these compounds and reinforce concerns about their toxicological relevance.

Overall, the widespread detection of QACs in wastewater, sediments, and indoor environments highlights their persistence, mobility, and bioactivity, warranting closer examination of their toxicity and ecological impact.

### 2.4. Environmental Hazard

**Figure 4:**
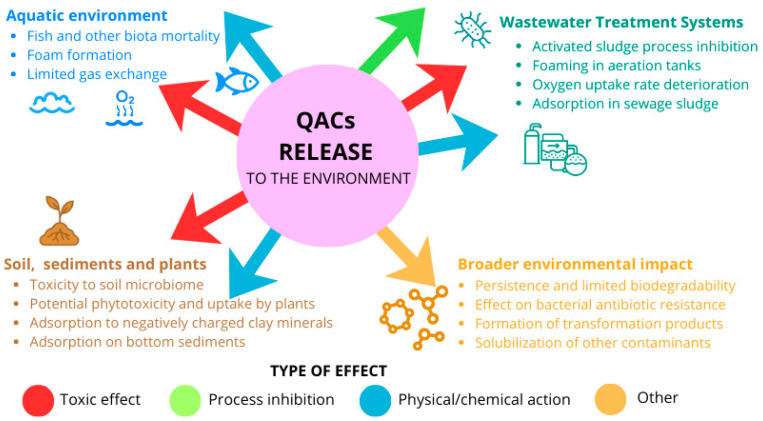
Overview of the environmental release and effects of QACs, including their impact on aquatic systems, soil and plants, wastewater treatment processes, and overall ecosystem persistence.

The frequent detection of cationic surfactants in industrial effluents and surface waters underscores their environmental significance. Once released into the aquatic environment, they interact with suspended solids, sediments, and biota, which not only promotes their accumulation but also amplifies their potential toxicity (Figure 4). These interactions form the basis for understanding their environmental hazard. CSs affect a wide range of physical and biological processes. By reducing the surface tension of water, they limit the diffusion of atmospheric oxygen and hence the efficiency of aerobic biodegradation. Surfactants have been shown to significantly reduce oxygen mass transfer in aeration systems by modifying bubble dynamics and interfacial properties [53,54]. Reduced oxygen diffusion is a major concern in aerobic wastewater treatment, as aeration can account for up to 60% of total operating costs [55].

Surfactants alter interfacial properties and promote the dispersion of colloidal and micellar aggregates, thus increasing turbidity and reducing the penetration of light into surface waters. This, in turn, suppresses photosynthesis and the growth of aquatic plants and algae, leading to oxygen depletion and further deterioration of water quality. Another undesirable surfactant action is increased foaming of water bodies and changes in various physicochemical properties of the soil [56].

The amphiphilic structure of surfactants facilitates the solubilization of hydrophobic organic contaminants [57], thereby increasing their mobility and bioavailability in aquatic environments. By forming micelles and mixed aggregates, surfactants improve the apparent solubility of compounds such as pesticides, phenols, and pharmaceuticals [58,59,60,61,62,63,64,65,66,67,68]. Although cationic surfactants are less prone to complex with metal cations, their adsorption on suspended solids and sediments can alter surface charges, indirectly influencing the mobility of heavy metals [60].

The strong biocidal properties of QACs underpin their broad use as disinfectants, sanitizers, and preservatives. However, the same mechanism that makes them potent antimicrobial agents also renders them hazardous when released into the environment uncontrolled. Numerous studies have demonstrated that QACs exert toxic effects on a wide range of aquatic organisms, including fish, daphnids, algae, rotifers, and microorganisms essential for biological wastewater treatment [61,62,63]. As summarized in Table 5, effective concentrations for aquatic species and wastewater microorganisms are typically below 1 mg/L, classifying QACs as very toxic to aquatic life according to the UN Globally Harmonized System [64]. In addition, several QACs have been shown to interfere with seed germination and inhibit the growth of higher plants [45,65].

In the context of wastewater treatment, in addition to their negative impact on oxygen uptake efficiency [71], QACs are known to interfere with key microbial processes responsible for pollutant removal. For instance, BAC concentrations of 2 mg/L or higher can inhibit nitrification and biological nutrient removal [72,73], with complete inhibition of nitrification occurring at 15 mg/L [74,75]. For COD utilization and denitrification, the reported inhibition values were 14.9 mg QAC/L and 0.27 mg QAC/L, respectively [75,76].

QACs also affect anaerobic digestion of waste activated sludge. He et al. [77] reported that BAC concentrations greater than 8 mg/g of TSS inhibited methanogenesis (inhibition ratios of 84.7%, 74.1% and 66.0% for C_12_, C_14_ and C_16_ within a 5-day test), caused short-chain fatty acids, and altered the microbial composition by reducing *Firmicutes*, *Bacteroidetes*, and *Chloroflexi*. Complete inhibition of methanogenesis has been reported to appear at QACs concentration of 100 mg/L [78,79].

Finally, the environmental appearance of QACs has been associated with the promotion of bacterial antibiotic resistance, as these compounds can enhance the transfer and persistence of antibiotic resistance genes (ARGs) within microbial communities, posing a serious risk to both ecosystems and human health [80,81,82].

## 3. Conventional Treatment Technologies for Cationic Surfactant Removal

Although cationic surfactants form a chemically diverse group, their behaviour during treatment is governed by a limited set of recurring phenomena: strong adsorption to solids, pronounced surface activity, charge and the ability to form micelles or mixed aggregates. These features fundamentally shape how QACs interact with unit operations in wastewater treatment plants.

**Figure 5:**
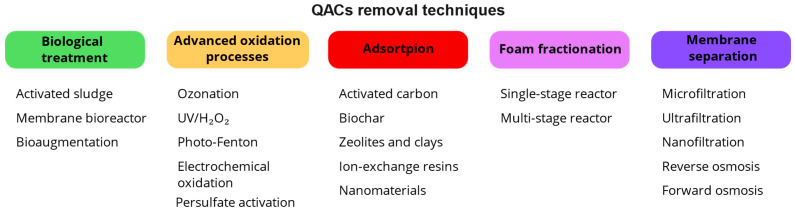
Processes applied for QACs removal from aqueous solutions.

The following subsections critically evaluate conventional approaches, outlining their removal mechanisms, practical performance ranges, operational constraints, and suitability for different wastewater types. Before examining individual processes in detail, Figure 5 summarises the main treatment routes that have been reported for surfactant-containing wastewaters. In the following sections, the discussion is therefore limited to the most relevant and well-established approaches, while methods of marginal importance or highly specific configurations are included in the diagram only for contextual completeness.

### 3.1. Foam Fractionation

Foam fractionation is a separation technique that utilizes the strong surface activity of surfactants. The method is based on the bubbling of air through contaminated water, where amphiphilic molecules adsorb at the air–water interface. Bubbles transport surfactants to the surface, concentrating surfactants in the foam, which can then be collected and removed [82].

The foaming process shows promise for treating industrial wastewater with high concentrations of surfactants, such as effluents from the textile, laundry and pharmaceutical sectors. However, scalability, foam stability, and the handling of the concentrated foam phase remain challenges for practical implementation. This process has long been applied in mineral processing and in the recovery of proteins or biosurfactants, but its application to wastewater treatment has gained attention in recent years. Foam fractionation is particularly attractive for the removal of cationic surfactants, which typically exhibit strong surface activity and readily partition into the foam. Moreover, when combined with other contaminants (e.g., PFAS), cationic surfactants such as CTAB or CTAC have also been tested as cofoaming agents to enhance removal efficiency through the formation of cation–anion complexes in solution and increased surface adsorption [83].

Boonyasuwat et al. [84] investigated the removal of the cationic surfactant cetylpyridinium chloride (CPC) at a concentration of 0.25 CMC (CMC = 322 mg/L) using a four-stage foam fractionation reactor. The air flow was kept constant at 100 L/min. The CPC removal efficiencies were 20%, 24%, 68% and 70% for the one-, two, three- and four-stage processes, respectively. Kishor Kumar [85] investigated the possibility of CTAB recovery (initial concentration 5 times CMC) using foam reactor. Authors demonstrated that 65% recovery ratio may be achieved, however the process performance was significantly limited by the presence of dissolved salts, which compromise foam stability and reduce separation efficiency. Scalability and the handling of the concentrated froth also remain challenges for practical implementation.

### 3.2. Chemical Coagulation and Flocculation

Coagulation–flocculation is a widely applied process for the removal of suspended solids, colloids, and organic micropollutants [86,87,88], including anionic surfactants. For example, Aboulhassan et al. [89] reported that coagulation with ferric salts (dose 900 mg L^−1^) was highly effective in the treatment of industrial wastewater from a microelectronics factory (initial concentration 935.45 ± 17.31 mg L^−1^ anionic surfactants), achieving up to 99% removal efficiency.

The primary mechanisms underlying coagulation and flocculation include electrical double layer compression, charge neutralization, bridging, and sweeping [90]. However, the efficiency of this process for cationic surfactants is fundamentally limited. Due to their permanent positive charge, QACs do not interact electrostatically with positively charged coagulant species (Al^3+^/Fe^3+^), making charge neutralization ineffective. Instead, removal can occur only through non-specific mechanisms such as sweep flocculation (in which contaminants are entrapped in precipitating hydroxide flocs) [90] or adsorption onto natural organic matter present in the water.

Moreover, the presence of CSs in treated solution may affect the efficacy of coagulation in terms of remaining pollutants removal. Vasiljević et al. [91] noted that the presence of surfactants can even destabilize coagulation efficiency, since amphiphilic molecules stabilize colloidal suspensions rather than promote their aggregation.

### 3.3. Biological Treatment

Biological treatment is a key process for the removal of cationic surfactants from municipal wastewater. Conventional activated sludge systems have been shown to achieve removal efficiencies exceeding 95% for quaternary ammonium compounds (QACs) under favorable operating conditions [92]. Nevertheless, reported efficiencies vary depending on influent characteristics and process conditions.

Although certain cationic surfactants are theoretically biodegradable [93], their removal in typical activated sludge systems occurs predominantly through rapid adsorption onto biomass rather than metabolic degradation [94,95]. This sorption significantly reduces the bioavailability of these compounds, thus inhibiting their biodegradation [96]. The detection of QACs in the sludge further suggests that biological treatment primarily represents a phase transfer process rather than true mineralization.

The adsorption of QACs onto activated sludge is governed by physicochemical and operational factors. The affinity of QACs to biomass increases with the length of the alkyl chain and the overall hydrophobicity of the molecule, which enhance van der Waals and hydrophobic interactions [97]. The negative surface charge and organic content of activated sludge facilitate electrostatic attraction, leading to the adsorption of cationic surfactants onto the flocs, while a higher biomass concentration and longer sludge retention time further improve the removal efficiency. On the contrary, high ionic strength, elevated temperature, and competition from other cations or organic matter can reduce the sorption capacity. Although QACs remain permanently charged over a wide pH range, the protonation of functional groups on the sludge surface may slightly affect the binding affinity under acidic conditions [39,65,97].

It should be noted that adsorption and biodegradation processes occur in biological systems simultaneously, making it challenging to assess their relative contributions to the overall removal of QACs. To separate the effects of adsorption from biodegradation, several studies have employed immobilized microbial systems or biofilm reactors, which reduce suspended solids and limit direct uptake by biomass [98,99]. This approach enables the evaluation of metabolic degradation under controlled conditions, without interference from sorptive removal. For example, Fortunato et al. [99] achieved a 99.3% removal of benzalkonium chloride in an aerobic biofilm upflow reactor, confirming that, under optimized conditions, true biodegradation of QACs is possible. However, such conditions are rarely achieved in full-scale wastewater treatment plants, where adsorption remains the dominant mechanism governing the fate of QACs during biological treatment.

### 3.4. Adsorption and Ion Exchange

Adsorption represents one of the most effective and widely applied physicochemical methods for the removal of various micropollutants from aqueous solutions [100,101,102,103]. Due to their amphiphilic character and permanent positive charge, QACs exhibit a strong affinity for negatively charged surfaces.

A wide range of adsorbents have been tested for the removal of QACs, including activated carbon [104], zeolites [105,106], clays [107,108,109], and biochar-based materials [110]. In recent years, increasing attention has been paid to low-cost and naturally available adsorbents, e.g., agricultural waste–derived sorbents [111,112], or low-cost household materials [113]. Among these, activated carbon remains the most widely used due to its high surface area, porosity, and surface functionality, enabling more than 90% removal of major QACs (BAC, CTAB, and DDAC) under optimized conditions [114,115,116].

The removal mechanism involves both electrostatic and hydrophobic interactions, enabling the binding of molecules within the porous structure of the sorbent. This dual mechanism was clearly demonstrated by Kim et al. [114], who tested powdered activated carbon for the removal of BAC_12_, BAC_14_, and BAC_16_ homologues in batch experiments. They reported that BACs with longer alkyl chains were adsorbed more effectively onto PAC than those with shorter chains, suggesting that van der Waals and hydrophobic forces play a dominant role in the adsorption of BAC_14_ and BAC_16_, whereas electrostatic interactions primarily govern the adsorption of BAC_12_.

The pore size distribution of the adsorbent plays a crucial role in QACs removal. At low concentrations, QAC molecules are primarily adsorbed in micropores, whereas at higher concentrations, meso- and macropores also contribute [115,117]. Tanada et al. [115] reported that activated carbon with the highest micropore fraction (<20 Å) and surface area (1150 m^2^/g) exhibited the greatest adsorption capacity, increasing from 38 to 265 mg/g as the BAC concentration rose from 1 to 1000 mg/L.

The adsorption efficiency is also affected by pH, ionic strength, and competing ions. Although the QACs remain permanently charged, the sorbent surface becomes less negative at low pH, weakening the electrostatic attraction. The increased ionic strength and multivalent cations (Ca^2+^, Mg^2+^) compress the electrical double layer and reduce adsorption, while dissolved organic matter can compete for active sites.

The ionic nature of CSs makes them particularly suitable for removal by ion exchange, which often provides selectivity and efficiency that are higher than those of conventional adsorption. In comparison to physical sorption, ion exchange offers stronger and more specific electrostatic interactions with functional groups of the exchanger, allowing effective separation even at low contaminant concentrations. Effective removal of QACs, such as BAC and CTAB, has been demonstrated to be achieved using synthetic cation exchange resins, particularly those based on polystyrene–divinylbenzene. A strongly acidic resin, applied at a dose of 20 mL/L and a contact time of 2 h, reduced surfactant concentrations from 1000 mg/L to 23 mg/L for BAC and 246 mg/L for CTAB [118].

An advantage of ion exchange over conventional adsorption is the possibility of resin regeneration and reuse [119]. In a paper by Kaleta et al. [120] it was demonstrated that even after several cycles, the ion-exchange capacity decreases by only about 10%, indicating that both exchange and physical sorption occur simultaneously, resulting in a higher overall removal efficiency compared to traditional adsorbents. However, the main drawback of ion exchange is the formation of toxic regeneration effluents containing concentrated brine and QACs residues, a chemically aggressive mixture that poses a serious environmental challenge.

### 3.5. Advanced Oxidation Processes (AOPs)

Advanced oxidation processes (AOPs) represent a broad group of treatment technologies designed to generate highly reactive oxygen species, primarily hydroxyl and sulfate radicals (•OH, SO_4_•^−^). These chemicals are capable of non-selectively oxidizing a wide spectrum of persistent organic contaminants [121]. Their exceptionally high redox potential (E°(•OH) = 2.8 V; E°(SO_4_•^−^) = 2.6 V) allows the degradation of compounds resistant to conventional treatment methods, including pharmaceuticals [122], pesticides [123], dyes [124] and surfactants [125].

AOPs provide a distinct approach compared to conventional treatment, aiming not only to transfer QACs to another phase (as occurs in adsorption or coagulation) but also to chemically destroy their molecular structure. The degradation mechanisms of QACS include mainly the removal of hydrogen, hydroxylation, and cleavage of the C-N bond [126,127].

On this basis, several AOP strategies have been tested for the degradation of QACs. Although initial studies focused on standalone oxidants such as ozone or UV light, enhanced processes, ex. UV-activated persulfate, UV/H_2_O_2_, and VUV/UV-C systems have proven substantially more effective, achieving faster reaction kinetics. Huang et al. [128] investigated the degradation of BAC species during ozonation. Stand-alone ozonation removed 71% of the compound within 40 min (initial BAC concentration 38.6 mg/L, ozone dose 38 mg/L-gas, pH 6.2). The presence of free chlorine (10 mg/L) further enhanced the process, increasing the removal efficiency to 91% under the same reaction time. Ultraviolet combined with persulfate (UV/PS) treatment was introduced for dodecyltrimethylammonium chloride (DTAC) removal from model solutions [129]. Both •OH, SO_4_•^−^ radicals were shown to participate in the degradation process. While direct UV photolysis and persulfate alone were ineffective (removal ~10%), the UV/PS system achieved 91% DTAC removal in 10 min contact time (75.6 μM PS; 870 mJ/cm^2^).

Fenton-based systems represent another promising strategy. The degradation of cationic surfactant CTAB by the Fenton process was studied by Huang et al. [130]. The authors reported 90% removal within 30 min process (optimal process parameters: m(H_2_O_2_)m/(CTAB) 1: 2.3; H_2_O_2_ dosage 8.0 mg/L; Fe^2+^ concentration 20 mg/L; pH 3.0). Dong et al. [131] applied a heterogeneous Fenton system in which iron–carbon–bentonite–alginate beads (Fe/C-BABs) served as the immobilised iron source for •OH generation. Under optimal conditions (pH 3.0, 60 mM H_2_O_2_, 50 g/L catalyst), the process achieved 87% removal of BAC while maintaining minimal iron leaching (0.07%).

Although AOPs are highly effective in degrading QACs, the formation and fate of their transformation products (TPs) remain considerably understudied. Most available studies focus on overall removal efficiency or mineralisation proxies (TOC, COD), while only a limited number provide structural identification of intermediates, which requires advanced analytical techniques such as GC–MS or high-resolution MS. A detailed investigation of BAC transformation products was provided by Zhang et al. [132], who identified ten intermediates formed during Fenton oxidation. The detected TPs included benzyl-dimethylamine, dimethylamine, benzoic, and p-hydroxybenzoic acids, as well as long-chain aldehydes and fatty acids such as dodecanal and dodecanoic acid. These results confirm that BAC degradation follows parallel pathways involving debenzylation, dealkylation, and oxidation of the alkyl chain, while the aromatic ring remains largely intact. Hong et al. [133] showed that the transformation products formed during PS/Fe^2+^ oxidation of BAC were markedly less toxic than the parent compound. According to microbial fuel cell tests, the strong inhibitory effect of BAC was substantially reduced after oxidation, indicating that cleavage of the benzyl–ammonium structure not only drives degradation, but also leads to clear detoxification of the mixture.

### 3.6. Comparative Assessment

Despite the wide range of conventional technologies available for wastewater treatment, their performance for the removal of QACs remains highly variable. Classical methods such as coagulation–flocculation or adsorption are mainly based on phase-transfer mechanisms (entrapment, sorption, association with solids) rather than chemical transformation of QACs. Consequently, they provide only partial removal and do not degrade the parent compounds. Even more advanced biological systems, including activated sludge, eliminate QACs predominantly through sorption to biomass, with little or no biodegradation of disinfectant-type QACs (e.g., BAC, DDAC, CPC and CTAB). Although AOPs are highly effective and often achieve near-complete degradation of QACs, the formation and behaviour of intermediate transformation products remain poorly understood. Only a few studies have identified these species, and their toxicity or persistence is largely unknown. This uncertainty highlights the need to combine AOPs with other treatment steps rather than relying on oxidation alone.

The effectiveness of these methods also strongly depends on the type of wastewater. High-strength industrial or textile effluents behave differently than municipal or hospital wastewater, and elevated salinity, organic load, or co-occurring surfactants can further hinder removal efficiency. To highlight the practical relevance and limitations of each approach, Table 6 summarizes the key mechanisms, typical removal efficiencies, and the suitability of individual conventional methods for different wastewater matrices.

The next section focusses on membrane-based processes, which remove QACs and their potential oxidation by-products through physical separation and offer a complementary, non-destructive alternative to chemical oxidation.

## 4. Membrane-Based Purification Techniques

Membrane processes are key unit operations for the treatment of solutions containing cationic surfactants, due to their high efficiency and application potential (Table 7). In contrast to adsorption, coagulation, or biological treatment, membrane processes provide a true barrier effect, physically retaining surfactant species instead of transferring them into another phase. One of the most important factors that influence the efficiency of surfactant removal in these processes is the critical micelle concentration (CMC) and the size of the micelles formed.

Low-pressure membrane processes, such as microfiltration (MF) and ultrafiltration (UF), are relatively effective for removing CSs from solutions with concentrations above the CMC (c > CMC). In this concentration range, surfactant molecules form micelles (Figure 1) that are sufficiently large to be retained by the relatively wide pores of the MF and UF membranes.

In contrast, when CSs are predominantly in monomeric form (c < CMC), high-pressure membrane processes, such as nanofiltration (NF) and, in some cases, reverse osmosis (RO), are required. These membranes have smaller pore sizes and higher rejection capacities, enabling effective separation of individual surfactant molecules that cannot be retained by low-pressure membranes [134]. This distinction underscores the importance of relating surfactant concentration to the CMC when selecting an appropriate membrane process, as well as the way micelle formation, membrane pore size, and operating pressure influence removal efficiency.

The main limitations of membrane processes for the separation of cationic surfactants from aqueous solutions include two phenomena: declines in hydraulic performance as a result of membrane fouling and the permeation of monomeric surfactant molecules into the permeate, which can occur even in micellar solutions.

For example, in paper [135] CTAB solutions (CMC ≈ 350 mg/L, micelle diameter ≈ 3.5 nm) at concentrations ranging from 0.025 CMC to 3 CMC were treated using two nanofiltration membranes (polyethersulfone; pore radius 0.80–1.29 nm and 0.59–0.93 nm). The overall removal of surfactant did not exceed 80% even at the highest concentrations, indicating that monomer permeation occurred above the CMC. Moreover, application of tighter reverse osmosis membranes (polyamide, NaCl rejection = 99.8% and cellulose acetate NaCl rejection = 98.0%) for CTAB solutions treatment (initial concentration 25, 100, 1000, 5000, 10,000) representing both monomeric and micellar forms did not bring complete CTAB removal. Reported rejections ranged from 96 to 99% for the PA membrane and from 92 to 99% for the CA membrane, demonstrating that even RO membranes allow limited monomer [136].

**Figure 6:**
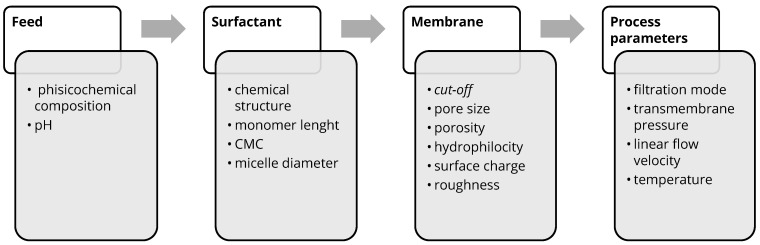
Parameters affecting membrane-based surfactant removal.

The intensity of membrane fouling and the selectivity of the membrane process are determined by a combination of the following factors: physicochemical properties of the feed solution, surfactant properties, membrane material properties and membrane process operational parameters (Figure 6).

Studies in fouling area [137,138] indicate that the mechanism of membrane fouling by surfactants is highly complex and has not yet been fully elucidated. However, its intensity is dependent on the form of surfactant occurrence (monomers vs. micelles), the pore size of the membrane and its surface characteristics (hydrophilicity, surface charge). Research on membrane blocking [137] did not allow the identification of a single dominant fouling mechanism; instead, the results indicate that fouling arises from the simultaneous penetration and adsorption of monomeric surfactant molecules inside membrane pores and the formation of a micelle-rich surface layer (cake/gel layer) on the membrane.

Table 8 provides a consolidated overview of membrane performance under various operating conditions reported in the literature both for model solutions and real wastewater. As shown, removal efficiencies and fouling behaviour differ substantially between MF, UF, NF, RO, and FO, largely depending on the concentration of the surfactant, the complexity of the matrix, and the membrane characteristics. This comparison emphasises that membrane selection must be closely aligned with surfactant speciation (monomers vs. micelles) and wastewater composition, and that optimal treatment often requires multi-stage or hybrid configurations.

Forward osmosis (FO) has emerged as a highly promising technique for concentrating and recovering cationic surfactants, particularly quaternary ammonium compounds (QACs), by using the osmotic gradient between a feed solution and a draw solution to induce water transport across a semipermeable membrane. Operating at low or zero hydraulic pressure, FO minimises fouling and energy consumption while preserving surfactant integrity, making it attractive for industrial applications.

Klimonda & Kowalska [142] demonstrated that FO can achieve nearly complete separation of QACs over a wide concentration range (50–1000 mg/L) without detectable leakage into the draw solution. In their experiments, BAC and CTAB concentrations increased from 508 and 488 mg/L to 974 and 980 mg/L, respectively, corresponding to an approximate twofold reduction in feed volume. No BAC contamination of the draw solution was observed, and CTAB penetration was minimal, with only 2.2 mg/L detected at the end of the experiment (retention 99.8%), confirming FO’s effectiveness in concentrating and recovering QACs from model solutions.

Extending these findings to real industrial wastewater, FO was applied to the rinsing effluent from esterquat-based surfactant production, containing ~2500 mg/L cationic surfactants and a COD of ~20,000 mg O_2_/L [143]. Using a 1 M NaCl draw solution, the FO process reduced the volume of wastewater by 50%, which led to a twofold increase in COD and cationic surfactant concentration. The rise in feed solution salinity (67–612 mg Cl^−^/L) indicated the occurrence of reverse salt flux, while TOC levels in the draw solution reached approximately 200 mg/L, showing partial transfer of organic contaminants through the membrane. These findings confirm that FO is effective for wastewater concentration and promote circular economy approaches by supporting water reuse and reducing freshwater demand.

## 5. Conclusions

Among the wide range of investigated technologies, biological processes, advanced oxidation, and membrane-based methods currently demonstrate the greatest potential for the removal of quaternary ammonium compounds (QACs). Nevertheless, their performance remains highly dependent on operational conditions and wastewater characteristics, which limits their universal applicability. Conventional treatment technologies provide only partial removal, relying mainly on sorption, phase transfer, or foam enrichment, and often generate secondary waste streams. Advanced oxidation processes enable efficient degradation but require high operational input, and the formation and behaviour of transformation products remain insufficiently understood. Pressure-driven membrane methods provide an effective physical barrier but are often limited by the permeation of low-molecular-weight monomers and by severe fouling, strongly influenced by surfactant speciation, concentration, and matrix complexity. No single technology ensures a comprehensive removal of QACs; therefore, effective treatment requires tailored, multi-step, or hybrid approaches. Future research should prioritise integrated processes, improved membrane materials, and the systematic evaluation of transformation products to meet increasingly stringent regulatory requirements.

## Figures and Tables

**Table 1:** Molecular characteristics of selected surfactants, including monomer molecular weight, aggregation number (n_a_g), and the resulting micelle molecular weight.

Surfactant	Type	Molecular Weight of Monomer, Da	Aggregation Number, n_a_g	Molecular Weight of Micelle, Da	Ref.
SDS (sodium dodecyl sulfate)	AS	288.5	62	18,000	[6]
CPC (cetylpyridinium chloride)	CS	358	95	34,010	[7]
CTAB (cetyltrimethylammonium bromide)	CS	364.45	75	27,370	[8]
TX-100 (Triton X-100)	NS	625	140	87,500	[7]

**Table 2:** Overview of the main applications of cationic surfactants.

Surfactant Type	Applications
Classical QACs (e.g., BAC, CPC, CTAB)	1. Healthcare and Personal Care - Disinfectants, preservatives, and antiseptics in hospitals, clinics, households, and cosmetic products - Active ingredients in soaps, hand sanitizers, wet wipes, and surface cleaners - Preservatives in ophthalmic and nasal formulations - Active ingredients in lozenges, throat sprays, and toothpastes - Antiviral agents against SARS-CoV-2 2. Laboratory and Research Applications - Agents in DNA extraction buffers and protein electrophoresis systems - Cleaning and disinfecting laboratory equipment and surfaces 3. Industrial and Environmental Applications - Algaecides and biocides in swimming pool and spa maintenance products - Sanitizers in food-processing and dairy plants - Disinfectants and biocides in water treatment systems and industrial equipment
Esterquats (e.g., TEAQ, DEEDMAC)	1. Textile and Laundry Applications - Fabric softeners and textile conditioners in household and industrial laundry products - Antistatic agents for natural and synthetic fibers 2. Automotive Care Applications - Antistatic and gloss-enhancing agents in car care products (e.g., waxes, interior cleaners, dashboard sprays) 3. Personal Care and Cosmetic Applications - Conditioning agents in hair and skin care formulations - Emulsifiers in cosmetic creams and lotions

**Table 3:** Concentrations of quaternary ammonium compounds (QACs) in various wastewater sources, including hospital, municipal, domestic, laundry and industrial effluents.

Source of Wastewater	Surfactant	Concentration	Comments	Ref.
Hospital	QACs	5 mg/L	ΣCSs	[39]
Hospital	BAC	2.8 mg/L	Homologue included: C_12_	[40]
Hospital	BAC	2.8 mg/L	Homologue included: C_12_	[38]
Municipal wastewater influents	BAC	0.8–170 µg/L	Homologues included: C_12_–C_18_	[41]
	DDAC	n.d.–200 µg/L	Homologues included: C_10_–C_16_	
	ATAC	0.7–27 µg/L	Homologues included: C_12_–C_16_	
Domestic	QACs	0.5 mg/L	ΣCSs	[39]
Laundry	BAC	2.1 mg/L	Homologue included: C_12_	[40]
Laundry	TEAQ	29.8 ± 7.9 mg/L	Textile rising cycle wastewater	[42]
Industry	CSs	780 mg/L	ΣCSs in mixed wastewater from technological park	[36]
Industry (dairy)	BAC	20 µg/L	Homologue included: C_12_	[39]

n.d.—not detected, BAC—benzalkonium chloride, DDAC—didecyldimethylammonium chloride, ATAC—alkyltrimethylammonium chloride, TEAQ triethanolamine esterquat.

**Table 4:** Occurrence of quaternary ammonium compounds (QACs) in wastewater, sludge, sediments, and other environmental matrices.

Medium	Location/Study	ΣQACs Concentration	Dominant Compound	Ref.
WWTP influent	USA	31.9 µg/L	BAC C_14_ DDAC C_10_ ATMAC C_16_	[43]
WWTP effluents	USA, Minnesota (data from 12 WWTPs)	0.4–8.3 µg/L	BAC C_14_ DDAC C_10_ ATMAC C_16_	[45]
	USA, Northeastern (data from 3 WWTPs)	0.04–3.5 µg/L	BAC C_12_–C_16_	[44]
	USA, New York State	0.55 µg/L	BAC C_14_ DDAC C_10_ ATMAC C_16_	[43]
WWTP sludge	Austria	2–103 µg/g dw (Σ12 QACs)	BAC C_12_–C_14_ DDAC C_10_–C_18_	[40]
	Germany (Bad Nauheim, Hesse)	94.6 µg/g dw (Σ18 QACs)	BAC C_12_ (30%) DADMAC C_10_ (25%)	[46]
Lake sediments	USA, Lake Winona (receiving WWTP discharges)	2.4–4.9 µg/g dw	DADMAC	[45]
River sediments	USA, New York City (estuarine sediments)	1–114 µg/g dw	ATMAC	[45]
	China, Pearl River (estuary)	0.2 µg/g dw	BAC C_12_ ATMAC C_18_	[47]
	Austria, Danube, Ybbs, Liesing, Schwechat	6–3600 µg/g (median values typically 20–600 µg/g)	BAC C_12_–C_14_ DDAC C_10_–C_18_	[40]
Soil	China, Chongqing	0.0047–0.22 µg/g (average ≈ 0.06 µg/g)	BAC C_12_ ATMAC C_14_	[48]
	Germany, Lahn river area (foodplain solids)	0.007–2.1 µg/g	DADMAC C_18_	[49]
Dust from homes and public areas	Chima, Shenzhen	13.8–150 µg/g (median values typically 42.2 µg/g)	-	[50]
Surface water	USA, New York State, Hudson and Mohawk Rivers	0.05 µg/L	DDAC C_18_	[51]
	USA, New York State	0.02 µg/L	DDAC C_18_	[51]
Seawater	England, Tynemouth	0.12–0.27 µg/L	DADMAC C_10_	[52]

BAC—benzalkonium chloride, DDAC—didecyldimethylammonium chloride, ATMAC—alkyltrimethylammonium chlorides, DADMAC—diallyldimethylammonium chloride.

**Table 5:** Toxicity data for representative quaternary ammonium compounds (QACs) toward selected aquatic organisms.

Organism/ Test Species	QAC	Endpoint	Effect Concentration	Ref.
Algae				
*Pseudokirchneriella subcapitata*	BAC	72 h EC_50_	41–255 µg/L	[38]
		72 h EC_50_	255 µg/L	[66]
	DDAC	72 h EC_50_	21 µg/L	[38]
Invertebrates				
*Daphnia magna*	BAC	48 h EC_50_	38.2 µg/L	[67]
		48 h EC_50_	16 µg/L	[68]
*Ceriodaphnia dubia*	BAC	24 h LC_50_	404 µg/L	[67]
*Caenorhabditis elegans*	BAC	24 h LC_50_	3.1 mg/L	[69]
*Phaeodactylum* *tricornutum*	CTAB	72 h-IC_50_	0.554 mg/L	[70]
Vertebrates				
*Danio rerio*	CPC	120 h EC_50_	175.9 µg/L	[61]
*Bombina orientalis*	CPC	168 h LC_50_	697 µg/L	[70]

BAC—benzalkonium chloride, DDAC—didecyldimethylammonium chloride, CTAB—Cetyltrimethylammonium bromide, CPC—Cetylpyridinium chloride.

**Table 6:** Comparative overview of conventional technologies used for QACs removal, highlighting removal mechanisms, performance ranges, and practical applicability to industrial, municipal, and hospital wastewater.

Process	Mechanism	Typical QACs Removal	Industrial Wastewater (High Concentrations of QACs)	Municipal Wastewater (Low Concentrations of QACs)	Hospital Wastewater (Moderate Concentrations of QACs)	Comments
Coagulation–flocculation (Al/Fe)	Sweep flocculation; physical entrapment	Low (10–20%)	+ only for foam/turbidity; not QACs	+	+	Limited effectiveness because positively charged QACs are not neutralised by Al/Fe coagulants; removal occurs only via non-specific sweep flocculation.
Foam fractionation	Interfacial Adsorption	Moderate–high (50–90%)	+++ excellent for surfactant-rich effluents	+ limited foaming	+	Highly efficient for streams rich in surfactants, but performance decreases with high ionic strength.
Adsorption on activated carbon (GAC/PAC)	Hydrophobic sorption; micelle uptake	Moderate–high (40–90%)	+++ especially for long-chain QACs	+++	+++	Efficient for long-chain QACs, though adsorption is reduced by competing organics and high salinity; regeneration may be costly.
Ion exchange	Electrostatic binding, physical binding	High (80–99%)	+++	+	++	Very high removal; generates toxic brine regeneration effluents containing concentrated QACs, which require further treatment.
Biological treatment (activated sludge)	Sorption + biodegradation (esterquats)	Variable (50–95%)	− (microorganism inhibition)	++	+ risk of toxicity peaks	Removal driven mainly by sorption rather than biodegradation; risk of microbial inhibition at elevated QACs levels.
AOPs	Generation of highly reactive; non-selective chemical oxidation and breakdown of QAC structure	Very high (90–99%)	+++	+++	+++	Highly effective but requires strict pH/oxidant control and relatively high energy or reagent input, making operation costly; TPs may still require post-treatment.

The symbols (+/++/+++/–) indicate the qualitative applicability of each treatment to different wastewater types: +++ = highly effective, ++ = effective, + = limited effectiveness, and – = poor or unsuitable performance.

**Table 7:** Applicability of membrane processes for QAC removal.

Membrane Process	Type	Pore Size, nm	Dominant Mechanism	Applicability
MF	pressure-driven	100–10,000	Size exclusion	High concentrations (micellar solutions)
UF		2–100		
NF		0.5–2	Size and charge exclusion	Low concentrations (monomer solutions)
RO		<0.5		
FO	osmotically driven	<0.5		

**Table 8:** Comparison of pressure driven membrane processes for QACs removal.

Membrane Process Ref.	Conditions	Removal Efficiency/Limitations
UF and NF [139]	- model solutions, BAC 50–1000 mg/L - CMC = 350 mg/L - polymeric modules: ESP04 (4 kDa), AFC30 (0.2 kDa), AFC40 (0.3 kDa), and AFC80 (0.2 kDa) - crossflow mode	NF BAC removal >74% across feed concentrations of 50–1000 mg/L using NF (AFC40) Increasing BAC concentration considerably reduced permeate flux (fouling) Fouling depends on pore size, polymer hydrophilicity, and membrane charge The lowest MWCO (pore size) shows the least flux decline and a higher fouling resistance UF Lower retention of BAC compared to NF Pore blocking is the dominant fouling mechanism; UF is more prone to fouling Significant flux decline observed at higher BAC concentrations
UF and MF [140]	- model solutions, TEAQ 50–1000 mg/L - CMC = 0.026 mg/L - ceramic modules: 150 kDa, 0.14 μm and 0.45 μm - crossflow mode	Fouling was observed, especially in modules with smaller pores Modules with larger pores (0.45 μm) were more resistant to pore blocking Higher cross-flow velocity helps reduce blocking
NF [138]	- model solutions, Cetrimide 40 mg/L - CMC = 1320 mg/L - polymeric flat-sheet membranes: NF270 (155 Da), Desal51HL (190 Da), NTR7450 (310 Da), NFPES (1200 Da) - crossflow mode	High rejection is possible with low MWCO (89–97% for NF270 and Desal51HL) Fouling and separation efficiency was influenced by both pore size and surface charge The increase in electrostatic attraction resulted in more pronounced fouling and lower separation
UF and NF [141]	- wastewater generated during the washing of disinfectant production lines - COD 2000 mg O_2_/L, TOC 850 mg C/L and BAC 300 mg/L - polymeric modules: ESP04 (4 kDa) and AFC30 (0.2 kDa)	~30% BAC removal from wastewater when using only ESP04 or AFC30 modules Matrix complexity (other organics, high COD) strongly reduces membrane process effectiveness Significant flux decline observed due to fouling in real wastewater
MF + NF [42]	- cationic surfactants (esterquats) rich laundry wastewater - COD 80 mg O_2_/L, ΣCSs 30 mg/L, TOC 51 mg/L and turbidity 46 NTU - sequential treatment with two modules: MF (ceramic 0.45 µm) and NF (polymeric, 0.5 nm) - crossflow mode	Permeate suitable for landscape irrigation and toilet flushing Complete CSs removal in a two-step (MF + NF) process; other organics present in permeate (e.g., fragrances; TOC = 11 mg/L) Strong flux decline in the NF module
MF and UF [37]	- industrial wastewater from a surfactant manufacturing plant - with high surfactant concentration (~2668 ± 301 mg/L) - COD 20,800 ± 2800 mgO_2_/L, TOC 15,100 ± 200 mg C/L, ΣCSs 2700 ± 300 mg/L - ceramic modules: 0.45 µm, 0.14 µm, 150 kDa	0.45 µm module (MF) Moderate TEAQ retention and effective concentration Strong pore blocking and rapid flux decrease Monomeric surfactant may pass through 0.14 µm module (MF) High removal of organics and good retention of surfactant aggregates Cake-layer formation at higher concentrations of TEAQ Fouling sensitive to TMP and flow conditions 150 kDa module (UF) Very high TEAQ retention and efficient concentration Intense fouling due to adsorption and pore blocking Significant flux decline
NF [135]	- moles solutions, CTAB 0.025 CMC—3 CMC (9–1050 mg/L) - CMC ≈ 350 mg/L - micelle diameter ≈ 3.5 nm - polymeric flat-sheet membranes (PES; pore radius 0.80–1.29 nm and 0.59–0.93 nm).	CTAB removal did not exceed 80% even at the highest concentrations, Monomer permeation occurred above the CMC Fouling occurred; the higher surfactant concentration, the more pronounced fouling
RO [136]	- model solutions, CTAB 10–10,000 mg/L - CMC = 346 mg/L - polymeric flat-sheet membranes: UTC-73 (PA, NaCl rejection = 99.8%;), AFC30 (0.2 kDa), AFC40 (0.3 kDa), and SC3000 (CA, NaCl rejection = 98.0%;) - dead-end mode	CTAB retention exceeding 90% Fouling resistance of the strongly hydrophilic CA membrane over the entire range of CTAB concentrations There are no apparent interactions between the surfactant and the CA membrane surface

## Data Availability

No new data were created or analyzed in this study. Data sharing is not applicable to this article.

## References

[1] Nishmitha P.S., Akhilghosh K.A., Aiswriya V.P., Ramesh A., Muthuchamy M., Muthukumar A. (2025). Understanding emerging contaminants in water and wastewater: A comprehensive review on detection, impacts, and solutions. J. Hazard. Mater. Adv..

[2] Directive (EU) 2024/3019 of the European Parliament and of the Council of 27 November 2024 Concerning Urban Wastewater Treatment (Recast) (Text with EEA Relevance). https://eur-lex.europa.eu/eli/dir/2024/3019/oj/eng.

[3] Shaban S.M., Kang J., Kim D.H. (2010). Surfactants: Recent advances and their applications. Compos. Commun..

[4] Sheng J.J. (2010). Modern Chemical Enhanced Oil Recovery: Theory and Practice.

[5] De S., Mondal S. (2012). Micellar Enhanced Ultrafiltration: Fundamentals and Applications.

[6] Product Catalogue: Sodium Dodecyl Sulfate (28364); Thermo Fisher Scientific Inc.: Waltham, MA, USA, 2025. https://www.thermofisher.com/order/catalog/product/28364.

[7] Huang J.H., Zhao Y., Zeng G.M., Peng L., Li X., Liu L.X., Li F., Shi L.X., Yuan F. (2015). Micellar-enhanced ultrafiltration for the solubilization of various phenolic compounds with different surfactants. Water Sci. Technol..

[8] Pisárčik M., Devínsky F., Pupák M. (2015). Determination of micelle aggregation numbers of alkyltrimethylammonium bromide and sodium dodecyl sulfate surfactants using time-resolved fluorescence quenching. Open Chem..

[9] Coltelli M.B., Serpico A., Domenech R., Tronch M., Galli C., Sonzini P., Escrivà-Cerdán C., Mastroianni S., Lazzeri A., Licursi D. (2025). Fatty Amines in Detergents and Cosmetics: Current State and Biocircular Perspectives. Cosmetics.

[10] Marzullo P., Gruttadauria M., D’Anna F. (2024). Quaternary Ammonium Salts-Based Materials: A Review on Environmental Toxicity, Anti-Fouling Mechanisms and Applications in Marine and Water Treatment Industries. Biomolecules.

[11] PubChem Cetyltrimethylammonium Bromide (CTAB). National Center for Biotechnology Information. https://pubchem.ncbi.nlm.nih.gov/compound/Cetyltrimethylammonium-bromide.

[12] PubChem Cetylpyridinium Chloride (CPC). National Center for Biotechnology Information. https://pubchem.ncbi.nlm.nih.gov/compound/Cetylpyridinium-chloride.

[13] PubChem Benzalkonium Chloride. National Center for Biotechnology Information. https://pubchem.ncbi.nlm.nih.gov/compound/Benzalkonium-chloride.

[14] Mohapatra S., Lin Y., Goh S.G., Ng C., You L., Tran N.H., Gin K.Y.H. (2023). Quaternary ammonium compounds of emerging concern: Classification, occurrence, fate, toxicity and antimicrobial resistance. J. Hazard. Mater..

[15] Osimitz T.G., Droege W. (2021). Quaternary ammonium compounds: Perspectives on benefits, hazards, and risk. Toxicol. Res. Appl..

[16] Takada I., Miyazaki A., Igarashi C., Yamawaki Y., Hayase A., Mori T., Sakai T. (2025). Study on the relationship between viral inactivation and alkyl chain length of benzalkonium chloride. PLoS ONE.

[17] Jacobs W. (1916). The bactericidal properties of the quaternary salts of hexamethylenetetramine: The problem of the chemotherapy of experimental bacterial infections. J. Exp. Med..

[18] Domagk G. (1935). Ein Beitrag zur Chemotherapie der bakteriellen Infektionen. Dtsch. Med. Wochenschr..

[19] (2006). Reregistration Eligibility Decision (RED) for Alkyl Dimethyl Benzyl Ammonium Chloride (ADBAC). EPA739-R-06-009 U.S. Environmental Protection Agency. https://nepis.epa.gov/Exe/ZyNET.exe/P1005J4P.TXT?ZyActionD=ZyDocument&Client=EPA&Index=2006+Thru+2010&Docs=&Query=&Time=&EndTime=&SearchMethod=1&TocRestrict=n&Toc=&TocEntry=&QField=&QFieldYear=&QField-Month=&QFieldDay=&IntQFieldOp=0&ExtQFieldOp=0&XmlQuery=&File=D%3A%5Czyfiles%5CIndex%20Data%5C06thru10%5CTxt%5C00000011%5CP1005J4P.txt&User=ANONYMOUS&Password=anonymous&SortMethod=h%7C-&MaximumDocuments=1&FuzzyDegree=0&ImageQuality=r75g8/r75g8/x150y150g16/i425&Display=hpfr&DefSeekPage=x&SearchBack=ZyActionL&Back=ZyActionS&BackDesc=Results%20page&MaximumPages=1&ZyEntry=1&SeekPage=x&ZyPURL.

[20] Smith J., Colgate-Palmolive Company (1978). Fabric Softener and Anti-Static Compositions. U.S. Patent.

[21] Mishra S., Tyagi V.K. (2007). Ester Quats: The Novel Class of Cationic Fabric Softeners. J. Oleo Sci..

[22] Yorgancioglu A., Onem E., Sabyrkhanova S. (2025). Production of an Esterquat-Based Novel Softening Agent and Its Impact on Leather and Textile Quality. ACS Omega.

[23] Wysocki M., Stachowiak W., Smolibowski M., Olejniczak A., Niemczak M., Shamshina J.L. (2024). Rethinking the Esterquats: Synthesis, Stability, Ecotoxicity and Applications of Esterquats Incorporating Analogs of Betaine or Choline as the Cation in Their Structure. Int. J. Mol. Sci..

[24] EPA List N: Disinfectants for Coronavirus (COVID-19). https://www.epa.gov/coronavirus-and-disinfectants/about-list-n-disinfectants-coronavirus-covid-19.

[25] Menger F.M., Littau C.A. (1991). Gemini-surfactants: Synthesis and properties. J. Am. Chem. Soc..

[26] Ahmady A.R., Hosseinzadeh P., Solouk A., Akbari S., Szulc A.M., Brycki B.E. (2022). Cationic gemini surfactant properties, its potential as a promising bioapplication candidate, and strategies for improving its biocompatibility: A review. Adv. Colloid. Interface Sci..

[27] Zana R. (2002). Dimeric (Gemini) Surfactants: Effect of the Spacer Group on the Association Behavior in Aqueous Solution. J. Colloid. Interface Sci..

[28] Menger F.M., Keiper J.S. (2000). Gemini Surfactants. Berichte Dtsch. Chem. Ges..

[29] Piecuch A., Obłąk E., Guz-Regner K. (2016). Antibacterial Activity of Alanine-Derived Gemini Quaternary Ammonium Compounds. J. Surfactants Deterg..

[30] Ermolaev V.V., Arkhipova D.M., Miluykov V.A., Lyubina A.P., Amerhanova S.K., Kulik N.V., Voloshina A.D., Ananikov V.P. (2022). Sterically hindered quaternary phosphonium salts (Qpss): Antimicrobial activity and hemolytic and cytotoxic properties. Int. J. Mol. Sci..

[31] Zhou X., Wang L., Han J. (2024). Multifunctional Profragrances: Hydrolyzable Quaternary Pyridinium Surfactants for Dual-Controllable Perfume Release. J. Ind. Eng. Chem. Res..

[32] Bhadani A., Misono T., Singh S., Sakai K., Sakai H., Abe M. (2016). Structural diversity, physicochemical properties and application of imidazolium surfactants: Recent advances. Adv. Colloid. Interface Sci..

[33] Jaan A., Ali S., Javed M., Haider A., Munawar K.S., Althobaiti S.A., Rehman M.U. (2024). Study of Newly Synthesized Pyridinium-based Cationic Surfactants for Drug Interaction and Antibacterial Activity. Proc. Pak. Acad. Sci. A.

[34] IndustryResearch.biz Quaternary Ammonium Compounds Market Report. https://www.industryresearch.biz/market-reports/quaternary-ammonium-compounds-market-106853.

[35] Grand View Research Esterquats Market Size, Share & Trends Analysis Report. https://www.grandviewresearch.com/industry-analysis/esterquats-market.

[36] Orlandi M., Filosa N., Bettone M., Fendrich M., Girardini M., Battistini T., Miotello A. (2019). Treatment of surfactant-rich industrial wastewaters with concentrated sunlight: Toward solar wastewater remediation. Int. J. Environ. Sci. Technol..

[37] Klimonda A., Kowalska I. (2021). Membrane technology for the treatment of industrial wastewater containing cationic surfactants. Water Resour. Ind..

[38] Kreuzinger N., Fuerhacker M., Scharf S., Uhl M., Gans O., Grillitsch B. (2007). Methodological approach towards the environmental significance of uncharacterized substances-quaternary ammonium compounds as an example. Desalination.

[39] Zhao M., Gao J., Liu Y., Wang Z., Wu Z., Zhang H., Zhang Y. (2023). Short-term stress of quaternary ammonium compounds on intracellular and extracellular resistance genes in denitrification systems. Chem. Eng. J..

[40] Martínez-Carballo E., González-Barreiro C., Sitka A., Kreuzinger N., Scharf S., Gans O. (2007). Determination of selected quaternary ammonium compounds by liquid chromatography with mass spectrometry. Part II. Application to sediment and sludge samples in Austria. Environ. Pollut..

[41] Clara M., Scharf S., Scheffknecht C., Gans O. (2007). Occurrence of selected surfactants in untreated and treated sewage. Water Res..

[42] Klimonda A., Kowalska I. (2025). Water Recovery from Laundry Wastewater by Integrated Purification Systems. Membranes.

[43] Mahony A.K., McNamara P.J., Arnold W.A. (2023). Quaternary Ammonium Compounds (QACs) in Wastewater Influent and Effluent Throughout the COVID-19 Pandemic. Environ. Sci. Technol..

[44] Hladik M.L., Gross M.S., Black G.P., Kolpin D.W., Masoner J.R., Phillips P.J., Bradley P.M., Smalling K.L. (2024). Temporal concentrations of Quaternary ammonium compounds in wastewater treatment effluents during the COVID-19 pandemic, 2020–2021. Chemosphere.

[45] Arnold W.A., Blum A., Branyan J., Bruton T.A., Carignan C.C., Cortopassi G., Datta S., Dewitt J., Doherty A.C., Halden R.U. (2023). Quaternary Ammonium Compounds: A Chemical Class of Emerging Concern. Environ. Sci. Technol..

[46] Heyde B.J., Barthel A., Siemens J., Mulder I. (2020). A fast and robust method for the extraction and analysis of quaternary alkyl ammonium compounds from soil and sewage sludge. PLoS ONE.

[47] Li X., Luo X., Mai B., Liu J., Chen L., Lin S. (2014). Occurrence of quaternary ammonium compounds (QACs) and their application as a tracer for sewage derived pollution in urban estuarine sediments. Environ. Pollut..

[48] Guo Z., Qin C., Zhang L. (2024). Distribution and Characterization of Quaternary Ammonium Biocides Resistant Bacteria in Different Soils, in South-Western China. Microorganisms.

[49] Lennartz S., Weber C.J., Siemens J., Mulder I. (2025). Legacy pollution of floodplain soils with quaternary ammonium compounds—Insights into vertical distribution, historical trends and suspected microplastic carriers. Environ. Pollut..

[50] Cheng Y., Liu C., Lv Z., Liang Y., Xie Y., Wang C., Wan S., Leng X., Hu M., Zheng G. (2024). High-Resolution Mass Spectrometry Screening of Quaternary Ammonium Compounds (QACs) in Dust from Homes and Various Microenvironments in South China. Environ. Sci. Technol..

[51] Li Z.M., Jeong H.H., Kannan K. (2025). A survey of 27 quaternary ammonium compounds in surface water, drinking water, stormwater runoff, swimming pool water, and rainwater from New York State, USA. Water Res..

[52] Bassarab P., Williams D., Dean J.R., Ludkin E., Perry J.J. (2011). Determination of quaternary ammonium compounds in seawater samples by solid-phase extraction and liquid chromatography–mass spectrometry. J. Chromatogr. A.

[53] Badmus S.O., Amusa H.K., Oyehan T.A., Saleh T.A. (2021). Environmental risks and toxicity of surfactants: Overview of analysis, assessment, and remediation techniques. Environ. Sci. Pollut. Res..

[54] Campbell K., Wang J. (2020). New insights into the effect of surfactants on oxygen mass transfer in activated sludge process. J. Environ. Chem. Eng..

[55] Liu C., Li S., Zhang F. (2011). The oxygen transfer efficiency and economic cost analysis of aeration system in municipal wastewater treatment plant. Energy Procedia.

[56] Arora J., Ranjan A., Chauhan A., Biswas R., Rajput V.D., Sushkova S., Mandzhieva S., Minkina T., Jindal T. (2022). Surfactant pollution, an emerging threat to ecosystem: Approaches for effective bacterial degradation. J. Appl. Microbiol..

[57] Wang P., Keller A.A. (2008). Soil particle-size dependent partitioning behavior of pesticides within water–soil–cationic surfactant systems. Water Res..

[58] Kuan W.H., Liu Y.J., Hu C.Y. (2020). Effects of surfactants on the degradation of diclofenac by manganese oxide. Int. J. Environ. Res. Public Health.

[59] Oleszczuk P., Xing B. (2011). Influence of anionic, cationic and nonionic surfactants on adsorption and desorption of oxytetracycline by ultrasonically treated and non-treated multiwalled carbon nanotubes. Chemosphere.

[60] Ilari R., Etcheverry M., Zenobi C., Zanini G. (2014). Effect of the surfactant benzalkonium chloride in the sorption of paraquat and cadmium on montmorillonite. Int. J. Environ. Health.

[61] Qiu X., Tengbe M.S., Xia X., Dong K., Chen C., Shi Y., Li M., Xu H., Wu X., Chen K. (2022). Impacts of Cetylpyridinium Chloride on the Survival, Development, Behavior, and Oxidative Stress of Early-Life-Stage Zebrafish (Danio rerio). Antioxidants.

[62] Roberts D., Roberts J., Hodges G., Gutsell S., Ward R.S., Llewellyn C. (2013). Aquatic toxicity of cationic surfactants to Daphnia magna. SAR and QSAR. Environ. Res..

[63] Kaczerewska O., Martins R., Figueiredo J., Loureiro S., Tedim J. (2020). Environmental behaviour and ecotoxicity of cationic surfactants towards marine organisms. J. Hazard. Mater..

[64] UNECE (2011). Globally Harmonized System of Classification and Labelling of Chemicals (GHS).

[65] Li Y., Zhou C., Wang S., Lin Q., Ni Z., Qiu H., Morel J.L., Qiu R. (2019). Phytotoxicity and oxidative effects of typical quaternary ammonium compounds on wheat (*Triticum aestivum* L.) seedlings. Environ. Sci. Pollut. Res..

[66] Elersek T., Ženko M., Filipič M. (2018). Ecotoxicity of disinfectant benzalkonium chloride and its mixture with antineoplastic drug 5-fluorouracil towards alga *Pseudokirchneriella subcapitata*. PeerJ.

[67] Lavorgna M., Russo C., D’Abrosca B., Parrella A., Isidori M. (2016). Toxicity and genotoxicity of the quaternary ammonium compound benzalkonium chloride (BAC) using Daphnia magna and *Ceriodaphnia dubia* as model systems. Environ. Pollut..

[68] Chen Y., Geurts M., Sjollema S.B., Kramer N.I., Hermens J.L.M., Droge S.T.J. (2014). Acurate toxicity of the cationi surfactant C12-benzalkonium in different bioassays: How test design affects bioavailability and effect concentrations Get access Arrow. Environ. Toxicol. Chem..

[69] Sreevidya V.S., Lenz K.A., Svoboda K.R., Ma H. (2018). Benzalkonium chloride, benzethonium chloride, and chloroxylenol—Three replacement antimicrobials are more toxic than triclosan and triclocarban in two model organisms. Environ. Pollut..

[70] Park C.J., Song S.H., Kim D.H., Gye M.C. (2016). Developmental and acute toxicity of cetylpyridinium chloride in Bombina orientalis (Amphibia: Anura). Aquat. Toxicol..

[71] Zhang C., Tezel U., Li K., Liu D., 2016Ren R., Du J., Pavlostathis S.G. (2011). Evaluation and modeling of benzalkonium chloride inhibition and biodegradation in activated sludge. Water Res..

[72] Chacón L., Arias-Andres M., Mena F., Rivera L., Hernández L., Achi R., Garcia F., Rojas-Jimenez K. (2021). Short-term exposure to benzalkonium chloride in bacteria from activated sludge alters the community diversity and the antibiotic resistance profile. J. Water Health.

[73] Conidi D., Andalib M., Andres C., Bye C., Umble A., Dold P. (2019). Modeling quaternary ammonium compound inhibition of biological nutrient removal activated sludge. Water Sci. Technol..

[74] Yang J., Tezel U., Li K., Pavlostathis S.G. (2015). Prolonged exposure of mixed aerobic cultures to low temperature and benzalkonium chloride affect the rate and extent of nitrification. Bioresour. Technol..

[75] Hajaya M.G., Pavlostathis S.G. (2012). Fate and effect of benzalkonium chlorides in a continuous-flow biological nitrogen removal system treating poultry processing wastewater. Bioresour. Technol..

[76] Hajaya M. (2021). Mitigating the Effects of Quaternary Ammonium Compounds on Biological Wastewater Treatment Systems during the COVID-19 Pandemic. Jordan J. Earth Environ. Sci..

[77] He Z.W., Liu W.Z., Tang C.C., Liang B., Guo Z.C., Wang L., Ren Y.X., Wang A.J. (2019). Performance and microbial community responses of anaerobic digestion of waste activated sludge to residual benzalkonium chlorides. Energy Convers. Manag..

[78] Flores G.A.E., Fotidis I.A., Karakashev D.B., Kjellberg K., Angelidaki I. (2015). Effects of Benzalkonium Chloride, Proxel LV, P3 Hypochloran, Triton X-100 and DOWFAX 63N10 on anaerobic digestion processes. Bioresour. Technol..

[79] Tezel U., Pierson J.A., Pavlostathis S.G. (2006). Fate and effect of quaternary ammonium compounds on a mixed methanogenic culture. Water Res..

[80] Li D., Gao J., Dai H., Wang Z., Duan W. (2020). Long-term responses of antibiotic resistance genes under high concentration of enrofloxacin, sulfadiazine and triclosan in aerobic granular sludge system. Bioresour. Technol..

[81] Zhang C., Cui F., Zeng G.-M., Jiang M., Yang Z.-Z., Yu Z., Zhu M.-Y., Shen L.-Q. (2015). Quaternary ammonium compounds (QACs): A review on occurrence, fate and toxicity in the environment. Sci. Total Environ..

[82] Sochacki M., Michorczyk P., Vogt O. (2025). Foam Fractionation as an Efficient Method for the Separation and Recovery of Surfactants and Surface-Inactive Agents: State of the Art. ACS Omega.

[83] Lee C.S., Venkatesan A.K. (2024). Cationic surfactant-assisted foam fractionation enhances the removal of short-chain perfluoroalkyl substances from impacted water. Chemosphere.

[84] Boonyasuwatm S., Chavadejm S., Malakulm P., Scamehornm J.F. (2003). Anionic and cationic surfactant recovery from water using a multistage foam fractionator. Chem. Eng..

[85] Kumar A.K., Rawat N., Ghosh P. (2020). Removal and recovery of a cationic surfactant from its aqueous solution by foam fractionation. J. Environ. Chem. Eng..

[86] Tarigan M., Raji S., Al-Fatesh H., Czermak P., Ebrahimi M. (2025). The Occurrence of Micropollutants in the Aquatic Environment and Technologies for Their Removal. Processes.

[87] Nam S.-W., Yoon Y., Chae S., Kang J.-H., Zoh K.-D. (2017). Removal of Selected Micropollutants During Conventional and Advanced Water Treatment Processes. Environ. Eng. Sci..

[88] Kim M.-K., Zoh K.-D. (2016). Occurrence and removals of micropollutants in water environment. Environ. Eng. Res..

[89] Aboulhassan M.A., Souabi S., Yaacoubi A., Baudu M. (2006). Removal of surfactant from industrial wastewaters by coagulation flocculation process. Int. J. Environ. Sci. Technol..

[90] Lucas M.S., Teixeira A.R., Jorge N., Peres J.A. (2025). Industrial Wastewater Treatment by Coagulation–Flocculation and Advanced Oxidation Processes: A Review. Water.

[91] Vasiljević S., Vujić M., Agbaba J., Federici S., Ducoli S., Tomić R., Tubić A. (2023). Efficiency of Coagulation/Flocculation for the Removal of Complex Mixture of Textile Fibers from Water. Processes.

[92] Östman M., Fick J., Tysklind M. (2018). Detailed mass flows and removal efficiencies for biocides and antibiotics in Swedish sewage treatment plants. Sci. Total Environ..

[93] Paun I., Mitru D., Covaliu C.I., Paraschiv G., Nechifor G., Mogą I.C., Datcu-Manea A., Niţă-Lazăr M. (2021). Biodegradation of anionic and cationic surfactants using bacterial strains from activated sludge. Int. J. Conserv. Sci..

[94] Bergero M.F., Lucchesi G.I. (2018). Degradation of cationic surfactants using immobilized bacteria: Its effect on adsorption to activated sludge. J. Biotechnol..

[95] Ismail Z.Z., Tezel U., Pavlostathis S.G. (2010). Sorption of quaternary ammonium compounds to municipal sludge. Water Res..

[96] Kickham P., Otton S.V., Moore M.M., Ikonomou M.G., Gobas F. (2012). Relationship between biodegradation and sorption of phthalate esters and their metabolites in natural sediments. Environ. Toxicol. Chem..

[97] Ren R., Liu D., Li K., Sun J., Zhang C. (2011). Adsorption of Quaternary Ammonium Compounds onto Activated Sludge. J. Water Resour. Prot..

[98] Larsson Y., Mongelli A., Kisielius V., Bester K. (2024). Microbial biofilm metabolization of benzalkonium compounds (benzyl dimethyl dodecyl ammonium & benzyl dimethyl tetradecyl ammonium chloride). J. Hazard. Mater..

[99] Fortunato M.S., Baroni S., González A.J., Álvarez Roncancio J.D., Storino A., Parise C., Planes E., Gallego A., Korol S.E. (2019). Biodegradation and Detoxification of Benzalkonium Chloride in Synthetic and Industrial Effluents in Upflow Biofilm Aerobic Reactors. Water Air Soil Pollut..

[100] Ogbeh G.O., Ogunlela A.O., Akinbile C.O., Iwar R.T. (2025). Adsorption of organic micropollutants in water: A review of advances in modelling, mechanisms, adsorbents, and their characteristics. Environ. Eng. Res..

[101] Tong Y., McNamara P.J., Mayer B.K. (2019). Adsorption of organic micropollutants onto biochar: A review of relevant kinetics, mechanisms and equilibrium. Environ. Sci. Water Res. Technol..

[102] Betsholtz A., Falås P., Svahn O., Cimbritz M., Davidsson Å. (2024). New Perspectives on the Interactions between Adsorption and Degradation of Organic Micropollutants in Granular Activated Carbon Filters. Environ. Sci. Technol..

[103] Kamińska G., Bohdziewicz J. (2016). Potential of various materials for adsorption of micropollutants from wastewater. Environ. Prot. Eng..

[104] Kaya Y., Gönder Z., Vergili I., Barlas H. (2008). Removal of cetyltrimethylammonium bromide and sodium dodecylether sulfate by granular activated carbon. J. Sci. Ind. Res..

[105] Spiridonov A.M., Sokolova M.D., Fedoseeva V.I., Nikiforov L.A., Okhlopkova A.A. (2021). Adsorption complexes ‘zeolite–cationic surfactant’: Properties and surface activity in a polymer composite material based on ultra-high-molecular-weight polyethylene. Mater. Today Chem..

[106] Schwuger M.J., von Rybinski W., Krings P. (1983). Adsorption of Cationic Surfactants on Zeolite A. Adsorption from Solution.

[107] Kozak M., Domka L. (2004). Adsorption of the quaternary ammonium salts on montmorillonite. J. Phys. Chem. Solids.

[108] Gürses A., Karaca S., Aksakal F., Açikyildiz M. (2010). Monomer and micellar adsorptions of CTAB onto the clay/water interface. Desalination.

[109] Silva R.P., Gois A.G.B., Ramme M.O., Castro Dantas T.N., Barillas J.L.M., Santanna V.C. (2021). Adsorption of cetyltrimethyl ammonium bromide surfactant for organophilization of palygorskite clay. Clay Miner..

[110] Antunes E., Vuppaladadiyam A.K., Sarmah A.K., Varsha S.S.V., Pant K.K., Tiwari B., Pandey A. (2021). Application of biochar for emerging contaminant mitigation. Adv. Chem. Pollut. Environ. Manag. Prot..

[111] Ersa N.S. (2021). Adsorption mechanism on surfactant removal using eggshell waste and rice straw as economically biosorbent. IOP Conf. Ser. Earth Environ. Sci..

[112] Hosseinnia A., Hashtroudi M.S., Pazouki M., Banifatemi M. (2006). Removal of surfactants from wastewater by rice husk. Iran. J. Chem. Eng..

[113] Bitay E., Csavdari A. (2023). Some Unmodified Household Adsorbents for the Adsorption of Benzalkonium Chloride—A Kinetic and Thermodynamic Case Study for Commercially Available Paper. Toxics.

[114] Kim T.K., Choe W.S., Kim T., Chae S.H., Hwang Y.S., Zoh K.D. (2022). Adsorption of benzalkonium chlorides onto powdered activated carbon: Mechanisms and detoxification. Environ. Eng. Res..

[115] Tanada M., Miyoshi T., Tanada S. (1991). Adsorption Removal of Benzalkonium Chloride by Granular Activated Carbon for Medical Waste Water Treatment. Asia Pac. J. Public Health.

[116] Krivova M.G., Grinshpan D.D., Hedin N. (2013). Adsorption of CnTABr surfactants on activated carbons. Colloids Surf. A Physicochem. Eng. Asp..

[117] Bindes M.M., Franco M.R. (2015). Surfactant removal from aqueous solutions onto activated carbon using UV spectroscopy. Desalin. Water Treat..

[118] Klimonda A., Kowalska I. (2021). Sequential process: Membrane filtration and ion exchange as an effective method for water solution purification containing cationic surfactants. Desalin. Water Treat..

[119] Kowalska I. (2012). Regeneration of ion-exchange resins used for the separation of anionic surfactants from aqueous solutions. Environ. Pollut. Control.

[120] Kaleta J., Papciak D., Puszkarewicz A. (2018). Using ion exchange process in removal of selected organic pollution from aqueous solutions. J. Ecol. Eng..

[121] Andreozzi R., Caprio V., Insola A., Marotta R. (1999). Advanced oxidation processes (AOP) for water purification and recovery. Catal. Today.

[122] Kanakaraju D., Glass B.D., Goh P.S. (2025). Advanced oxidation process-mediated removal of pharmaceuticals from water: A review of recent advances. Environ. Sci. Pollut. Res..

[123] Dagnew M., Xue Q., Zhang J., Wang Z., Zhou A., Li M., Zhao C. (2025). A Review of Various Advanced Oxidation Techniques for Pesticide Degradation for Practical Application in Aqueous Environments. Sustainability.

[124] Gallego-Ramírez C., Chica E., Rubio-Clemente A. (2022). Coupling of Advanced Oxidation Technologies and Biochar for the Removal of Dyes. Water.

[125] Zambrano-Aranea J., Arcentales-Dueñas S., Escala-Benites F., Flores-Manrique N., Mosquera-Romero S. (2024). Advanced oxidation processes by UV/H_2_O_2_ for the removal of anionic surfactants in a decentralized wastewater treatment plant in Ecuador. Water Sci. Technol..

[126] Xiao Z.Y., Huang N., Wang Q., Wang W.L., Wu Q.Y., Hu H.Y. (2022). Advanced oxidation of dodecyl dimethyl benzyl ammonium chloride by VUV/UV/chlorine: Synergistic effect, radicals, and degradation pathway. Sep. Purif. Technol..

[127] Carbajo J.B., Petre A.L., Rosal R., Berná A., Letón P., García-Calvo E., Perdigón-Melón J.A. (2016). Ozonation as pre-treatment of activated sludge process of a wastewater containing benzalkonium chloride and NiO nanoparticles. Chem. Eng. J..

[128] Huang N., Wang W.L., Xu Z.B., Lee M.Y., Wu Q.Y., Hu H.Y. (2020). A study of synergistic oxidation between ozone and chlorine on benzalkonium chloride degradation: Reactive species and degradation pathway. Chem. Eng. J..

[129] Lee M.Y., Wang W.L., Xu Zbin Ye B., Wu Q.Y., Hu H.Y. (2019). The application of UV/PS oxidation for removal of a quaternary ammonium compound of dodecyl trimethyl ammonium chloride (DTAC): The kinetics and mechanism. Sci. Total Environ..

[130] Huang H.-B., Yin P.-H., Zhao L. (2009). Research on the degradation of cationic surfactant CTAB by Fenton process in waste water. J. Jinan Univ. Nat. Sci. Med..

[131] Dong Z., Zhang Q., Hong J. (2018). Effective benzalkonium chloride degradation by Fenton oxidation with iron–carbon–bentonite–alginate beads. Desalin. Water Treat..

[132] Zhang Q., Xia Y.F., Hong J.M. (2016). Mechanism and toxicity research of benzalkonium chloride oxidation in aqueous solution by H_2_O_2_/Fe^2+^ process. Environ. Sci. Pollut. Res..

[133] Hong J.M., Xia Y.F., Zhang Q., Chen B.Y. (2017). Oxidation of benzalkonium chloride in aqueous solution by S2O82−/Fe^2+^ process: Degradation pathway, and toxicity evaluation. J. Taiwan Inst. Chem. Eng..

[134] Kowalska I. (2012). Dead-end and cross-flow ultrafiltration of ionic and non-ionic surfactants. Desalin. Water Treat..

[135] Klimonda A., Kowalska I. (2017). Application of nanofiltration membranes for removal of surfactants from water solutions. E3S Web Conf..

[136] Halleb A., Nakajima M., Yokoyama F., Neves M.A. (2024). Effect of Surfactants on Reverse Osmosis Membrane Performance. Separations.

[137] Klimonda A., Kowalska I. (2023). Surfactant fouling in pressure-driven membrane processes. Environ. Prot. Eng..

[138] Boussu K., Kindts C., Vandecasteele Van der Bruggen B. (2007). Surfactant fouling of nanofiltration membranes: Measurements and mechanisms. ChemPhysChem.

[139] Kowalska I., Klimonda A. (2018). Purification and concentration of surfactant solutions using tubular nanofiltration modules. Desalin. Water Treat..

[140] Klimonda A., Kowalska I. (2020). Separation and concentration of cationic surfactant solutions with the use of ceramic modules. Environ. Prot. Eng..

[141] Klimonda A., Kowalska I. (2025). Integrated Purification Systems for the Removal of Disinfectants from Wastewater. Membranes.

[142] Klimonda A., Kowalska I. (2024). Forward osmosis as an effective concentration method for cationic surfactant solutions. Gaz Woda Technol. Sanit..

[143] Klimonda A., Kowalska I. (2025). Concentrating esterquat-based surfactant wastewater via forward osmosis: A step toward reuse. Water Resour. Ind..

